# The Clustering of mApoE Anti-Amyloidogenic Peptide on Nanoparticle Surface Does Not Alter Its Performance in Controlling Beta-Amyloid Aggregation

**DOI:** 10.3390/ijms21031066

**Published:** 2020-02-05

**Authors:** Roberta Corti, Alysia Cox, Valeria Cassina, Luca Nardo, Domenico Salerno, Claudia Adriana Marrano, Natalia Missana, Patrizia Andreozzi, Paulo Jacob Silva, Francesco Stellacci, Roberta Dal Magro, Francesca Re, Francesco Mantegazza

**Affiliations:** 1 School of Medicine and Surgery, NANOMIB Nanomedicine Center, University of Milano-Bicocca, 20854 Vedano al Lambro (MB), Italy; r.corti9@campus.unimib.it (R.C.); alcox@tcd.ie (A.C.); valeria.cassina@unimib.it (V.C.); luca.nardo@unimib.it (L.N.); domenico.salerno@unimib.it (D.S.); claudia.marrano@unimib.it (C.A.M.); natalia.missana@unimib.it (N.M.); roberta.dalmagro@unimib.it (R.D.M.); francesca.re1@unimib.it (F.R.); 2 Department of Material Science, University of Milano-Bicocca, 20125 Milan, Italy; 3 IFOM−FIRC Institute of Molecular Oncology, IFOM−IEO Campus, 20139 Milan, Italy; patrizia.andreozzi@unifi.it; 4 Department of Chemistry “Ugo Schiff”, Università degli Studi di Firenze, 50019 Sesto Fiorentino, Italy; 5 Institute of Materials, École Polytechnique Fédérale de Lausanne, 1015 Lausanne, Switzerlandfrancesco.stellacci@epfl.ch (F.S.)

**Keywords:** amyloid-β, mApoE, AFM, gold nanoparticles

## Abstract

The deposition of amyloid-β (Aβ) plaques in the brain is a significant pathological signature of Alzheimer’s disease, correlating with synaptic dysfunction and neurodegeneration. Several compounds, peptides, or drugs have been designed to redirect or stop Aβ aggregation. Among them, the trideca-peptide CWG-LRKLRKRLLR (mApoE), which is derived from the receptor binding sequence of apolipoprotein E, is effectively able to inhibit Aβ aggregation and to promote fibril disaggregation. Taking advantage of Atomic Force Microscopy (AFM) imaging and fluorescence techniques, we investigate if the clustering of mApoE on gold nanoparticles (AuNP) surface may affect its performance in controlling Aβ aggregation/disaggregation processes. The results showed that the ability of free mApoE to destroy preformed Aβ fibrils or to hinder the Aβ aggregation process is preserved after its clustering on AuNP. This allows the possibility to design multifunctional drug delivery systems with clustering of anti-amyloidogenic molecules on any NP surface without affecting their performance in controlling Aβ aggregation processes.

## 1. Introduction

The aggregative behavior of amyloid-β (Aβ) peptide, which is considered by many as responsible for the development of Alzheimer’s disease (AD), has been extensively analyzed for many years. According to the amyloid cascade hypothesis, Aβ species, in particular the Aβ1-42 variant, aggregate and form fibrillar plaques, which account for the extensive neurodegeneration and memory loss associated with AD [1,2].

The “sink effect” theory posits that reduction of amyloid levels in the periphery could lead to a shift in equilibrium and subsequent clearance of soluble Aβ assemblies from the brain [3]. However, large Aβ fibrils are unable to cross the blood brain barrier (BBB), unlike the smaller but toxic Aβ oligomers that accumulate in the AD brain. To study the aggregation patterns of amyloid proteins (i.e., formation of water-soluble aggregates), as well as to monitor the disaggregation process of fibrils into smaller water-soluble aggregates, several biophysical techniques, capable of providing information about the various steps in Aβ aggregation, have been extensively applied. The most established technique has been light scattering, allowing determination of the size of aggregates between 10 nm and 1 µm as a function of time [4,5,6,7]. However, scattering is blind to aggregates smaller than about 10 nm. This implies that the earliest events in the aggregation process are perceived as a “lag time” in such measurements. Moreover, due to the low scattering signal, scattering measurements are hardly applicable on samples diluted below several micromoles/liter, while, in the case at hand, Aβ concentrations in the cerebrospinal fluid of AD patients are <100 nM [8,9], and Aβ has been demonstrated to effectively interfere with neuronal communication at similarly low amounts [10,11,12].

Atomic force microscopy (AFM) has been widely applied to image the aggregates with resolution of about 10 nm, more effective than optical imaging. AFM provides intricate detail of intra-fibril morphology [13,14,15,16] and can be used to determine concentration, temperature and environmental conditions at which fibrillation, along with production of pre-fibrillar aggregates and oligomers, takes place in vitro [17]. More recently, sub-nanometer patterns of peptide ensembles have been obtained by AFM, suggesting that this technique might, in the future, allow a detailed morphologic study of the smallest aggregates [18]. Complementary information can be extracted by applying techniques based on fluorescence analysis. Historically, fluorescence has had an ancillary role with respect to other biochemical and biophysical methods for Aβ aggregation detection and elucidation of its molecular mechanisms. Fluorescence studies have been mainly confined to in vivo or ex vivo plaque imaging [19,20] and assessment of their fibrillar nature using the Thioflavin T (ThT) assay [21], involving the selective staining of fibrils by the fluorescent dye ThT. However, we have recently demonstrated [22] that quantitative fluorescence analyses can overcome the concentration limits plaguing scattering methods, assuring the high statistical reliability typical of ensemble techniques and extreme sensitivity to formation of oligomeric species from monomers. However, morphological information is sacrificed in fluorescence assays. 

The ability to monitor the size, shape and evolution over time of Aβ aggregates is particularly helpful when identifying and validating new molecules acting on preformed Aβ aggregates to promote their disaggregation, thus boosting the clearance of small soluble peptide aggregates from the brain [23,24]. A powerful strategy for controlling and understanding amyloid protein aggregation is the modulation of protein self-assembly [25]. Here, we studied the effects of a synthetic trideca-peptide CWG-LRKLRKRLLR (mApoE), mimicking the sequence of the Aβ-binding site of apolipoprotein E [26], on Aβ aggregation. Apolipoprotein E (apoE) is associated with Aβ in senile plaques and it is also able to bind soluble Aβ peptide with high avidity [26].

This peptide has been shown to slow down Aβ aggregation in vitro and disrupt amyloid plaques in the brains of a rat model of AD when attached on liposomes surface [27]. It is postulated that mApoE-functionalized liposomes act by stimulating the sink effect from the peripheral circulation, drawing Aβ from the brain to the blood, while also crossing the BBB in small amounts to degrade fibrils into smaller components that can be cleared from the brain [28]. Recently many scientists investigated the possibility to conjugate anti-amyloidogenic molecules on nanoparticles (NP) to control Aβ aggregation processes. Here we functionalized gold nanoparticles (AuNP), known to cross the BBB in vitro in a transwell model [29], with mApoE in order to evaluated if the mApoE clustering on NP surface may affect its performance. 

In order to overcome the limitations afflicting the biophysical investigation approaches described above, we have combined AFM and fluorescence techniques to probe the effect of AuNP on Aβ aggregation and pre-formed Aβ fibril disaggregation in comparison to that of the free peptide and bare AuNP. Moreover, we describe semi-quantitative analysis of AFM images to evaluate the aggregation and disaggregation of fibrils by automatic image processing. 

## 2. Result

### 2.1. AuNP Characterization

**Figure 1:**
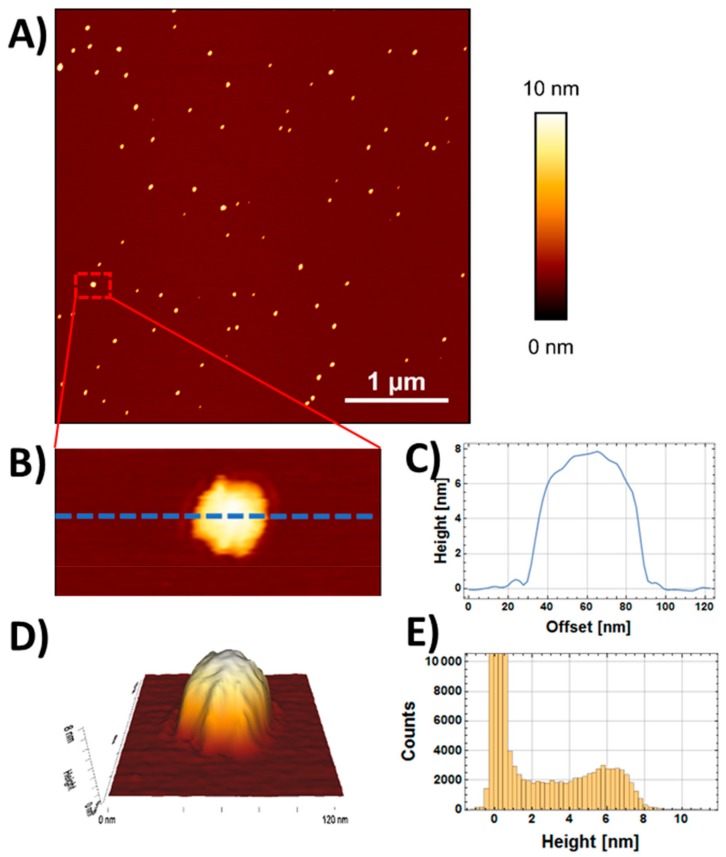
AFM characterization of non-functionalized gold nanoparticles (NP) on mica substrate. (**A**) Representative AFM image of AuNPs deposited on mica (4 × 4 µm^2^, 2048 × 2048 pixel, Z-scale 5 nm). (**B**) Digital zoom and section line. (**C**) Height profile obtained along the section shown in panel B. (**D**) 3D morphological reconstruction of a single AuNP obtained from the AFM measurements. (**E**) Statistical distribution of measured height of the image shown in panel A.

The morphology of the AuNPs was characterized both via AFM and Transmission Electron Microscopy (TEM) measurements. Figure 1A shows a representative AFM image of AuNPs deposited on mica. The dashed line in Figure 1B represents a section line along a single AuNP, while Figure 1C shows the resulting height profile. The relative 3D morphological representation, as obtained from AFM imaging, is shown in Figure 1D. In analogy with the approach of [30], the statistical distribution of measured height (Figure 1E) indicated that the maximum height was obtained for a value of 6.1 ± 0.5 nm. 

**Figure 2:**
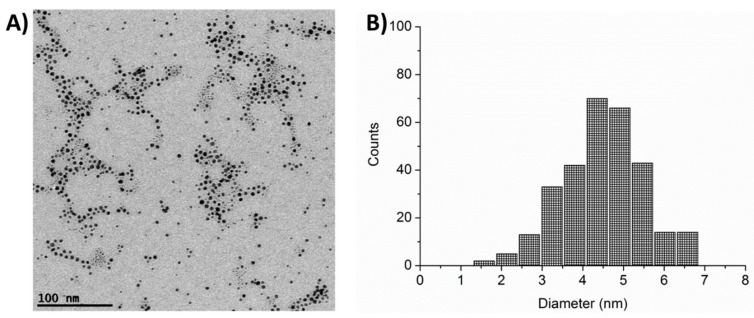
TEM characterization of non-functionalized AuNPs. (**A**) Representative TEM image of AuNPs. (**B**) Statistical distribution of AuNP particle diameter.

Analogous results were provided by TEM images, as reported in Figure 2, where a representative image (Figure 2A), together with the corresponding statistical distribution of the resulting diameters (Figure 2B), is shown. The diameter of the AuNPs obtained by analyzing TEM images analysis was 4.5 ± 0.6 nm, compatible with the AFM results, if we take into account that AFM will measure the gold core (imaged in TEM) plus the ligands whose extended length is about 1.4 nm but whose conformation onto a surface in dry state is basically unknown. 

The amount of mApoE bound to AuNP surface was 1.1 ± 0.3 μg peptide/μg Au as assessed by Bradford assay.

### 2.2. Effect of mApoE-Functionalized AuNP on Aβ Peptide Aggregation

From now on, all the results were obtained keeping constant the molar ratio between mApoE (free or bound to AuNP) and A*β* molecules. 

**Figure 3:**
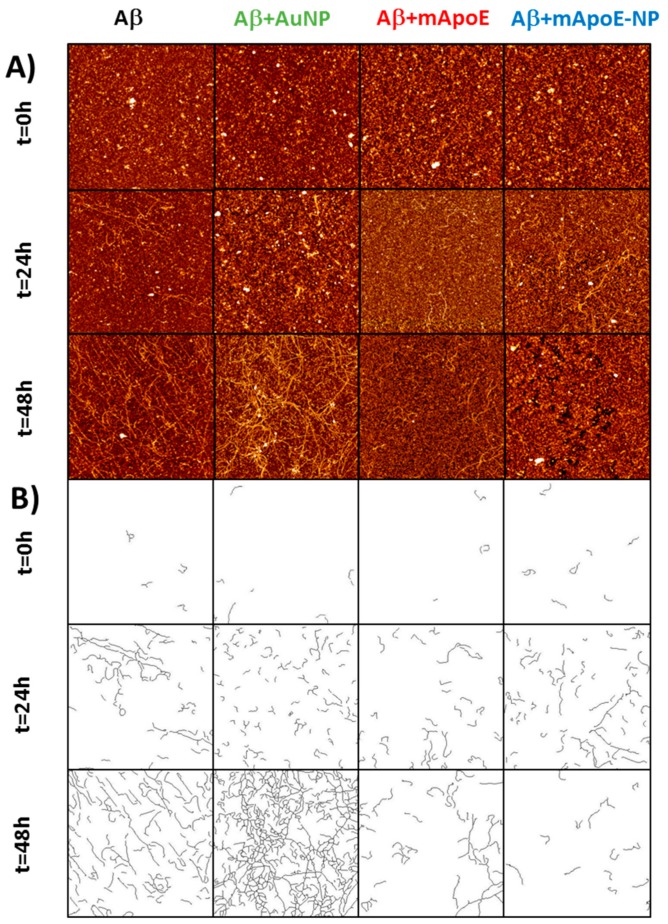
AFM imaging relative to the effect of external chemical agents (bare NP, free mApoE, and AuNP coated with mApoE) on the fibrillar aggregation of Aβ1-42. (**A**) Representative AFM images of typical aggregation patterns of bare Aβ1-42 amyloid fibrils (first column), of Aβ1-42 amyloid fibrils in presence of AuNP (second column), or free mApoE (third column), or AuNP coated with mApoE (fourth column). Data taken after incubation at 37 °C, at three different incubation times: *t* = 0 h (first line), *t* = 24 h (second line), and *t* = 48 h (third line). Images of 4 × 4 µm^2^, 1024 × 1024 pixel, Z-scale 10 nm. (**B**) Post-processed analysis of the AFM images reported in panel (**A**). The lines represent the resulting deposited fibrils as selected by the software procedure indicated in the Materials and Methods.

Figure 3A shows representative AFM images of different aggregation stages of Aβ, either alone or in the presence of free bare AuNP, mApoE, or mApoE-AuNP. The first column shows a progressive increase in the amount and length of deposited Aβ fibrils, changing from irregularly shaped objects at 0 h to dense bundles of long fibrils at 48 h. Fibrils were prepared in acidic environment and then used for all experiments in physiological buffer, and maintained their morphology, as described in reference [31]. The presence of bare AuNP (Figure 3A, second column) appeared to promote aggregation, with more abundant fibrils after 48 h. Conversely, the presence of either free mApoE (column 3) or mApoE-AuNP (column 4) clearly hindered the aggregation process, showing less concentrated and shorter fibrils at 48 h (Figure 3A). The mApoE-AuNP had similar efficacy with respect to free mApoE in limiting the Aβ aggregation. The process of fibril growth was quantitatively evaluated by measuring the number and the length of the deposited fibrils in the AFM images. 

In principle, the crowding of a fibrillary image could be estimated by considering the time evolution of the number of pixels above a fixed height threshold [32]. Unfortunately, by using this method it is not easy to obtain reliable conclusions for the images presented in Figure 3A due to excessive background noise. In order to corroborate the qualitative observations from Figure 3A, we applied an automatic algorithm allowing analysis of the images in order to measure length, width and number of deposited fibrils in each sample [33]. As described in the Material and Methods section, the procedure used involved binarizing each image above a brightness threshold, to remove smaller objects and to consider only elongated objects that can be classified as fibrils. This procedure is justified by the fact that the average length of Aβ fibrils is longer than small, spherically symmetric aggregates or debris. By visual inspection of the images, we estimated that the procedure eliminated the noise and preserved the elongated objects. Disregarding spherical objects, but not elongated aggregates, the algorithm recognized the fibrils in the AFM images and reported their width and length distributions, from which it was possible to extract the percentage of surface covered by fibrils. 

**Figure 4:**
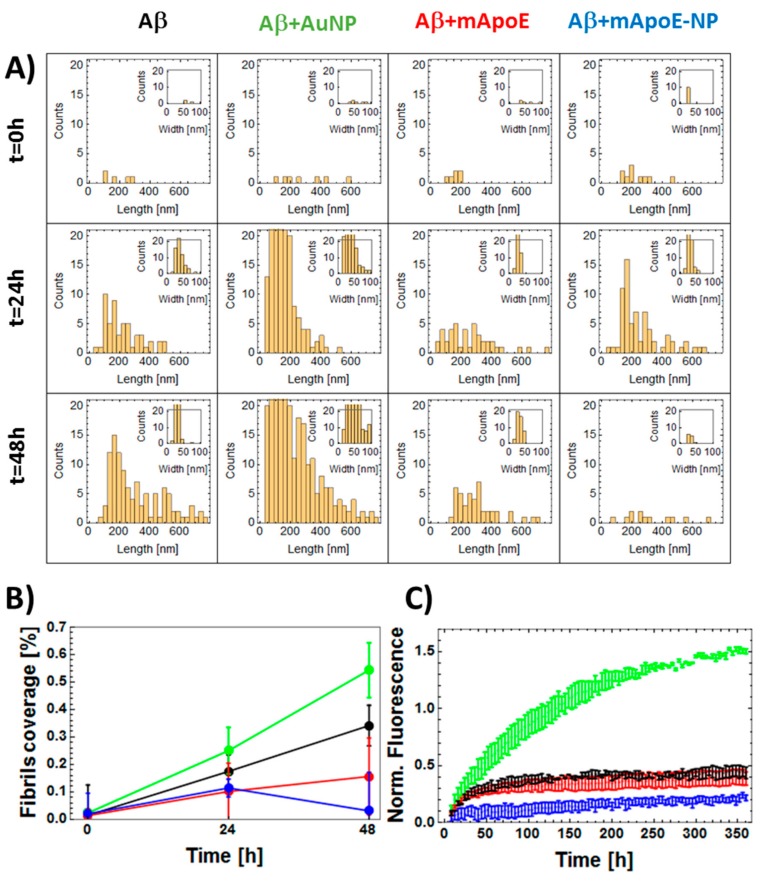
Quantitative analysis of the effect of external chemical agents on the aggregation of Aβ1-42. (**A**) Statistical distribution of fibrils length (main figures) and width (insets) as obtained from the analysis of AFM data reported in Figure 3B. Similar to Figure 3, data obtained using treatments indicated by the top labels (bare Aβ1-42, Aβ in the presence of bare AuNP, free mApoE, or AuNP coated with mApoE) and left labels reporting the different incubation times (*t* = 0 h, *t* = 24 h, *t* = 48 h). (**B**) Quantitative analysis of AFM images reported in panel A. Percentage of deposited fibrils covering the surface plotted as a function of time of aggregation. Data obtained for Aβ1-42 bare in solution (black dots) or in the presence of free AuNPs (green dots), free mApoE (red dots), or AuNP coated with mApoE (blue dots). (**C**) FITC fluorescence representing the aggregation of 30 nM FITC-Aβ1-42 as a function of the aggregation time: free Aβ (black dots) is compared to Aβ added with bare AuNP (green dots), free mApoE (red dots), or AuNPs coated with mApoE (blue dots). The normalized fluorescence values are obtained as (F(t) − F(0))/F(0), where F(t) and F(0) are the fluorescence values measured at time t and 0. The normalized fluorescence values are the average of three repetitions. Error bars represent standard error.

The results, confirming the trends inferred by visually inspecting the images of Figure 3A, are reported in Figure 3B and Figure 4A. Figure 3B shows the recognized fibrils and Figure 4A shows their length (main images) and width (insets) statistical distributions. In Figure 4B, the percentage of image surface covered by fibrils was plotted as a function of incubation time. This percentage was systematically larger in the presence of bare AuNP compared to Aβ alone, while both free mApoE and mApoE-AuNP were capable of reducing fibril formation. Despite the intrinsic AFM tip convolution effects that could broaden the lateral dimension, the width distribution of fibrils was almost constant (about 50 nm), indicating that the aggregation process was mainly longitudinal and not lateral (Figure 4A, insets). 

To further validate this result, the tendency of monomer-enriched preparations of FITC-Aβ to oligomerize was evaluated using the previously described fluorescence techniques [22]. The differential effects of adding bare AuNP, free mApoE, or mApoE-AuNP to Aβ on oligomer formation kinetics were probed. As FITC is covalently linked to the hydrophilic N-terminal of Aβ, oligomerization resulted in exposure of FITC to the solvent and a progressive increase in fluorescence (Figure 4C). The results showed that the normalized fluorescence values increased in time for all samples, with the largest increase in fluorescence after 6 h of Aβ aggregation in the presence of bare AuNP. A slower increase in fluorescence was detected with free mApoE and mApoE-AuNP, suggesting a lower final concentration of Aβ aggregates in these samples. 

### 2.3. Disaggregation Effect of mApoE-AuNP on Aβ Preformed Fibrils

**Figure 5:**
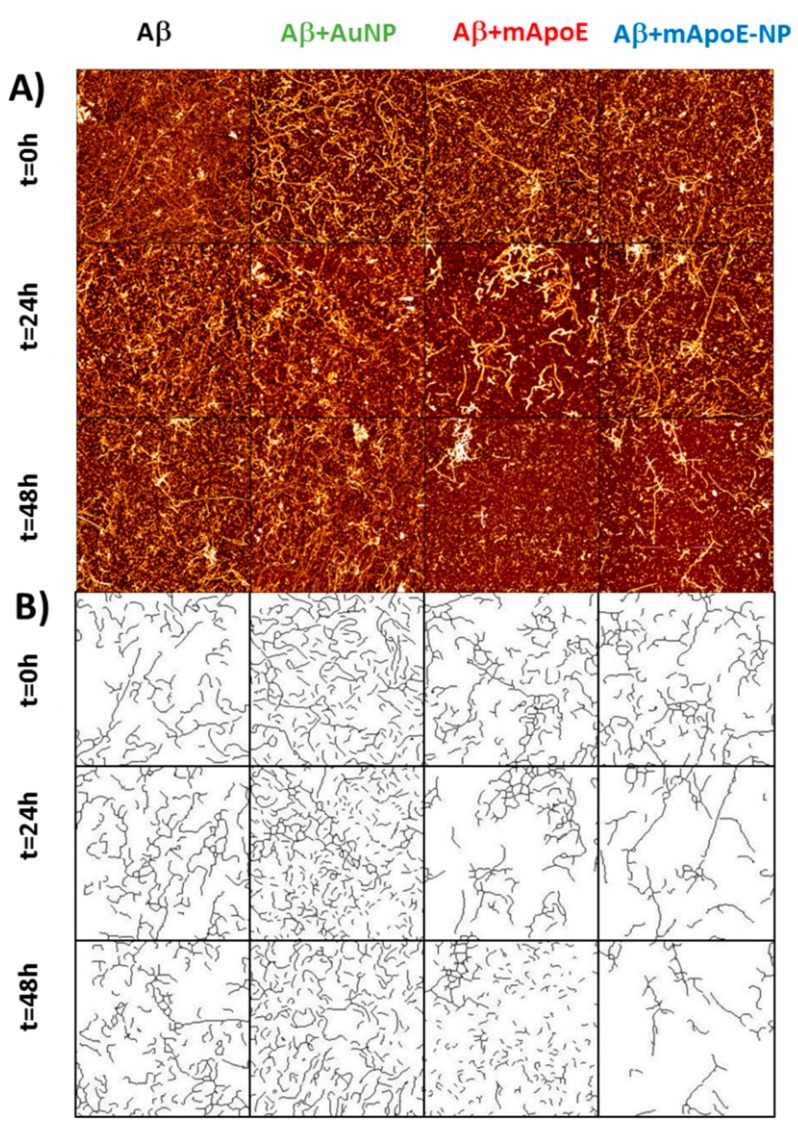
AFM imaging of the effect of external chemical agents (bare AuNP, free mApoE, and AuNP coated with mApoE) on the disaggregation of preformed Aβ fibrils. (**A**) Representative AFM images of typical disaggregation patterns of preformed Aβ1-42 amyloid fibrils (first column), of Aβ1-42 amyloid fibrils in presence of AuNP (second column), or free mApoE (third column), or AuNP coated with mApoE (fourth column). Data taken after incubation at 37 °C, at three different incubation times: *t* = 0 h (first line), *t* = 24 h (second line), and *t* = 48 h (third line). Images of 4 × 4 µm^2^, 1024 × 1024 pixel, Z-scale 10 nm. (**B**) Post-processed analysis of the AFM images reported in panel A. The lines represent the resulting deposited fibrils as selected by the software procedure indicated in the Materials and Methods.

**Figure 6:**
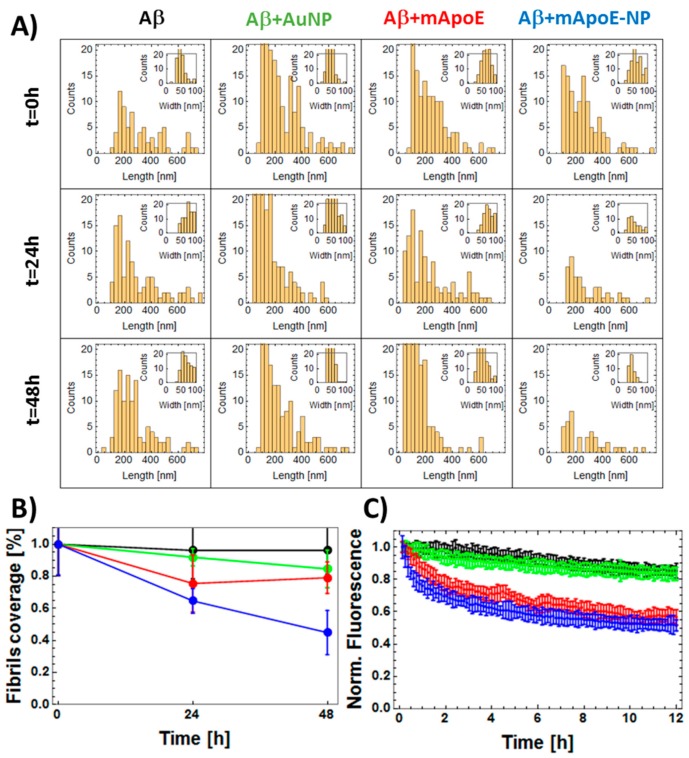
Quantitative analysis of the effect of external chemical agents on the disaggregation of preformed Aβ fibrils. (**A**) Statistical analysis of fibrils length (main figures) and width (insets) as obtained from the AFM data reported in Figure 5B. Similar to Figure 5B, data obtained following treatment as indicated by the top labels (bare Aβ1-42, Aβ in the presence of bare AuNP, free mApoE, or AuNP coated with mApoE) and left labels reporting the different incubation times (*t* = 0 h, *t* = 24 h, *t* = 48 h). (**B**) Quantitative analysis of AFM images reported in panel A. Normalized value of percentage of deposited fibrils plotted as a function of time of disaggregation. Data obtained for Aβ bare in solution (black dots) and in the presence of bare AuNP (green dots), free mApoE (red dots), AuNPs coated with mApoE (blue dots). The reported coverage value of deposited fibrils is normalized with respect to the average initial value (*t* = 0). (**C**) ThT assay-based evaluation of the evolution of preformed fibrils. THT fluorescence plotted as a function of time in 2 μM concentrated pre-fibrillated samples of Aβ stained with 10 mM THT: free Aβ (black squares) is compared to Aβ added with bare AuNP (green circles), free mApoE (red diamonds), or AuNPs coated with mApoE (blue stars). The normalized fluorescence values are obtained as F(t)/F(0), where F(t) and F(0) are the fluorescence values measured at time t and 0. The normalized fluorescence values are the average of three repetitions. Error bars represent standard error.

The ability of mApoE to disassemble preformed fibrils was studied using AFM imaging and fluorescence spectroscopy (Figure 5 and Figure 6). Figure 5A shows representative AFM images of preformed Aβ fibrils alone (first column) or after different times of incubation (rows) with bare AuNP (second column), free mApoE (third column) or mApoE-AuNP (fourth column). The images were analyzed using the same approach described for Figure 3B and Figure 4A and are shown in Figure 5B. The statistical distributions of fibril length and width are shown in the main plots and insets of Figure 6A. Figure 6B shows the percentage of deposited fibrils, normalized with respect to the number of fibrils at the initial state. Both visual inspection of Figure 5A,B and the quantitative analyses reported in Figure 6A,B suggest that the number of deposited fibrils was essentially constant in time for Aβ alone, while the presence of free mApoE or mApoE-AuNP caused progressive disaggregation of the preformed fibrils.

The above results were qualitatively confirmed by using the ThT assay (Figure 6C). The fluorescence value as a function of time was approximately constant when Aβ was free in solution, and it progressively decreased in the presence of free mApoE, bare AuNP, and mApoE-AuNP (Figure 6C). 

## 3. Discussion

Even if there are some concerns about the key role of Aβ in the pathogenesis of AD due to the failure of clinical trials [34], the progressive production and the reduced clearance of Aβ and its different aggregates remain potential therapeutic targets for AD treatment. A powerful strategy for controlling and understanding amyloid protein aggregation is the modulation of protein self-assembly. Multiple molecules have been found to interact with Aβ peptide, controlling its aggregation and disaggregation processes [35], including a multifunctional NP that disassembles Aβ fibrils and hinders Aβ aggregation [36]. Among them, one of the most promising antagonists for Aβ fibrillation seems to be the synthetic trideca-peptide CWG-LRKLRKRLLR (mApoE), which mimics the sequence of the Aβ-binding site of Apolipoprotein E [22,37], attached to lipid-based NP (i.e., liposomes).

A deeper knowledge of the molecular interactions responsible for activity of molecules that interact with Aβ could aid the design of new AD therapeutic agents. In this context, the surface functionalization of NP with these molecules combined with the advantages offered by NP turns out to be a promising strategy. For this purpose, we investigate if the anti-aggregative properties of mApoE against Aβ are preserved after its deposition on the AuNP surface. To do this we combined two techniques, AFM imaging and fluorescence spectroscopy, to evaluate in vitro the effects of bare AuNP, free mApoE, and mApoE-AuNP on both the aggregation of monomer-enriched Aβ preparations and the disaggregation of preformed Aβ fibrils.

The chosen techniques are complementary and provide similar conclusions. Indeed, AFM offers visual insight at the single molecule level on the multiplicity of diverse supramolecular structures within the aggregates, while fluorescence provides average parameters. In addition, fluorescence assays are normally applied to large molecular ensembles and provide a reliable sampling of heterogeneous systems such as fibrillar aggregates. Notably, the fluorescence assay used here allows examination of the initial (pre-fibrillation) steps of amyloid aggregation, which are undetectable through AFM analysis. Thus, the combination of the two methods offers a complete panorama of the aggregation dynamics.

These techniques allowed us to show that the presence of bare AuNP increases the rate of Aβ aggregation. This is in agreement with previous studies indicating that AuNP can catalyze Aβ aggregation depending on their surface characteristics, possibly via a seeding mechanism [35]. However, functionalization of the AuNP with mApoE alters their interaction with Aβ. Our findings suggest that both free peptide and mApoE-AuNP hinder Aβ aggregation and disassemble preformed Aβ fibrils, as already described using liposomes functionalized with mApoE [38,39]. 

Thus far, there have been many efforts to develop various monovalent Aβ ligands such as curcumin, but multivalent ligands may enhance the binding affinity towards Aβ [40]. Recently, foldamer–dendrimer conjugates have been optimized to selectively recognize and bind Aβ1–42 and its assemblies, fine tuning the topology of the multivalent interaction, without affecting the aggregation/disaggregation process of Aβ [41]. Thus, our results show that it is possible to cluster a single Aβ-binding ligand on NP surface, without decreasing its Aβ-binding affinity and its anti-amyloidogenic activity. 

This has implications in the rational design of ligands for therapeutics for AD and other amyloidosis, opening the possibility to attach anti-amyloidogenic molecules on NP without affecting their performance in controlling Aβ aggregation processes. However, in the generation of new nanotherapeutics against Aβ assemblies, this issue should be considered and tested for each single anti-amyloidogenic molecule used [42]. 

## 4. Materials and Methods

### 4.1. AuNP Synthesis

AuNP were prepared as previously described [28]. Briefly, 1.2 mmol of HAuCl_4_ was dissolved in 200 mL of ethanol. Then, 1.2 mmol of the hydrophilic 11-mercapto-1-dodecanethiol/diphenyl thiol ligand (MUS) ligand (synthesized as previously described [26]) was added while stirring. A saturated ethanol solution of sodium borohydride was added dropwise over 2 h. The solution was stirred for 3 h and then left overnight at 4 °C. The product was washed 3−5 times by suspending and centrifuging (5500 rpm) it in methanol, ethanol and acetone and finally with DI-water using Amicon Ultra-15 centrifugal filter devices 10 k Nominal Molecular Weight Limit (NMWL), (Merck Life Science S.r.l., Milano, Italy). AuNP were suspended in Milli-Q water and stored at room temperature (RT) until use. Immediately before use, AuNP were sonicated for 10 min at RT and then filtered through 0.22 μm filter. 

To attach mApoE to the AuNP surface, AuNP (0.1 mg/mL) were incubated with 0.6 mg/mL of mApoE in Milli-Q o/n at 4 °C [43]. Samples were ultracentrifuged at 165,000 g at 4 °C for 40 min, and the pellet, containing AuNP-mApoE, was washed twice with Milli-Q to remove unbound peptide. Each step of washing was followed by ultracentrifugation [29]. Pellet was resuspended in 0.5 mL of Milli-Q, and the amount of mApoE bound to AuNP surface was quantified by Bradford protein assay. 

### 4.2. Preparation of Aβ Samples

In order to obtain monomer-enriched Aβ preparations, lyophilized recombinant human Aβ1-42 peptide (Sigma–Aldrich, Milano, Italy) was treated as previously described [31]. Briefly, the peptide (1 mg/mL) was solubilized in 1,1,3,3,3-hexafluoro-2-propanol (HFIP; Sigma–Aldrich), dried, resuspended in DMSO at a concentration of 5 mM, and bath sonicated for 10 min. 

### 4.3. Aβ Aggregation and Disaggregation Process

The preparation of stable Aβ fibrils was obtained following the procedure previously described [31,44], with minor modifications. 

For the aggregation experiments, 5 mM Aβ was diluted in 10 mM HCl to a final concentration of 100 µM and incubated at 37 °C in the presence of either mApoE-coated AuNP (mApoE-AuNP; Au 50 µg/mL, mApoE 60 µg/mL), free AuNP (50 µg/mL), or mApoE alone (60 µg/mL). 

For disaggregation experiments, a fibril-enriched preparation was produced by dissolving the 5 mM Aβ sample in DMSO to a final concentration of 220 μM in 10 mM HCl, and incubating at 37 °C for 72 h. The Aβ fibrils were subsequently diluted to 100 μM in NaCl 10 mM (pH 7) and incubated with either mApoE-coated NP (Au 50 µg/mL; mApoE 60 µg/mL), free NP (50 µg/mL of Au), or mApoE alone (60 µg/mL) at 37 °C. For both aggregation and disaggregation experiments, 1 µL of each sample was collected at three incubation times (0, 24, 48 h) for fibril characterization by AFM imaging.

### 4.4. Atomic Force Microscopy and Transmission Electron Microscopy Imaging

The AFM imaging procedure used to characterize Aβ fibrils has been previously described [45,46]. Briefly, 1 µL of each 100 μM Aβ sample was collected at three incubation times (0, 24, 48 h), diluted 1:10 in HCl 10 mM and incubated for 5 min on a freshly cleaved mica substrate. Similarly, 10 µL of AuNP 50 µg/µL in HCl 5mM was deposited on a freshly cleaved mica substrate. After incubation, samples were rinsed with MilliQ water and dried under a gentle nitrogen flow. Is worth to notice that other deposition procedures have been reported in literature [47]; however, the presented method allows a differential comparison between the various situations under study. Measurements were acquired using a Nanowizard II (JPK Instruments, Berlin, Germany) instrument operating in tapping mode in air. In tapping mode, one of the most common imaging modes in AFM, the cantilever is oscillated near its resonance frequency close to the surface with and amplitude of about 50 nm. The oscillations force the tip to tap the surface. This interaction causes a reduction of the amplitude. The closer the tip is to the surface, the bigger is the reduction. Thanks to an electronic feedback, the tip-sample distance is kept constant by keeping constant the amplitude of the oscillation. The height image is reconstructed by collecting the correction of the tip-sample distance at any time. RTESP-300 (Bruker, Camarillo, CA, USA) cantilevers were used (nominal tip radius of 8 nm, nominal force constant of 40 N/m, resonance frequency of 300 kHz). Since its very small nominal radius compared to the rip radius of other common probes (20–30 nm), this probe was selected in order to minimize the tip convolution effects, which intrinsically broadened the lateral dimension of the structures. Contrarily, the height measurements were not influenced by the geometry of the tip.

AFM images (4 × 4 μm) were acquired at 1 Hz scan rate and at least 1024 × 1024 pixel resolution. The AFM images were analyzed using the commercial JPK image processing software and then by a customized image-analysis software described in the following section. All data in this study were verified by sampling a wide range of areas over the sample surfaces. 

For transmission electron microscopy analysis of AuNP, normal and ultra-thin plasma-coated carbon films were used. Ten microliters of AuNP at 0.2 mg/mL was transferred to grids and incubated for 10 min. Transmission electron microscopy analysis was performed by using a JEOL JEM 1010 microscope (Nieuw-Vennep, The Netherlands) operating at an acceleration voltage of 100 kV. The images were acquired at CICbiomaGUNE, Maria de los Angeles Ramirez (Instituto de Nanosistemas, Universidad Nacional de San Martín (INS-UNSAM), Av. 25 de Mayo 1021, San Martín, Buenos Aires, Argentina).

### 4.5. Morphological Analysis of AuNP and Fibril Recognition

The analysis of AFM images of deposited AuNP was obtained by using Mathematica (Wolfram, Champaign, IL, USA) morphological characterization software. For Aβ fibril identification over the background noise, images were post-processed as previously described [33]. To obtain automated fibril detection from AFM images, we adapted a MATLAB code (Matlab, MathWorks Inc, Natick, MA, USA) capable of measuring both fibril length and width distributions [33]. Each image was analyzed in the context of an orientation map obtained through a five-step process: (i) fibril smoothing by coherence-enhancing anisotropic diffusion filtering; (ii) contrast enhancement by top hat filtering; (iii) binarization by thresholding (classifying which pixels belong to fibers and which do not); (iv) skeletonization of the fibers to single-pixel; (v) orientation mapping from the result of diffusion filtering. Though this method can be partially affected by an overestimation of the fibril width, it is a reliable, semi-quantitative method for characterizing the aggregation and disaggregation processes.

### 4.6. Evaluation of Aβ Aggregation Kinetics by Fluorescence Experiments

The oligomerization kinetics of Aβ alone or with bare AuNP, free mApoE, or mApoE-AuNP were studied with a previously described fluorimetric protocol [22] using FITC-Ala-Aβ1-42 (Bachem, Schwerte, Germany), which determines the initial stages of aggregation with a high degree of precision. Moreover, the aggregation extent of FITC-Ala-Aβ1-42 was comparable to unlabeled Aβ1-42 [22]. FITC-Ala-Aβ1-42 was enriched in monomers by suspending it at 20 µM in hexafluoroisopropanol, sonicating for 5 min and vigorously vortexing for 5 min. The solvent was then evaporated from the solution under nitrogen flux, and the obtained films were kept at −20 °C until use. Before experiments, FITC-Ala-Aβ1-42 was resuspended in DMSO at a concentration of 30 µM, and it was sonicated and stirred as described above to remove residual large aggregates. This solution was diluted 1:1000 in 0.1 × PBS (pH 7.4, ionic strength 15 mM to avoid NP aggregation) to a final concentration of 30 nM. AuNP and mApoE were added to a concentration of 15 ng/mL of Au and 18 ng/mL, respectively, in order to conserve the mApoE-to-Aβ1-42 stoichiometric ratio of 1:3 established for the AFM measurements. Fluorescence measurements were performed in parallel in a thermostatic water-bath at 25 °C using quartz cuvettes (Hellma, Jena, Germany) in a Cary Eclipse spectrofluorometer (Agilent, Santa Clara, CA, USA). The samples were excited at 485 nm and the FITC spectra recorded every 4 min for 6 h in the spectral region 500–700 nm. From each spectrum the fluorescence intensity value was measured at 517 nm (FITC emission peak). The relative normalized fluorescence value, f(t), was defined by the equation

f(t) = (F(t) − F(0))/F(0)(1)

where F(t) is the fluorescence detected at 517 nm extracted from the spectrum measured at t min after the beginning of the experiment plotted as a function of aggregation time. All measurements were repeated on three different solutions.

### 4.7. Evaluation of Aβ Fibril Disaggregation by Thioflavin T Fluorescence Assay

Preformed Aβ fibrils, as described above, were diluted to a peptide concentration of 2 µM in 0.1 × PBS with 10 µM freshly dissolved thioflavin T (ThT) dye (Merck Life Science S.r.l., Milano, Italy). ThT dye was prepared as a 10 mM stock in 50 mM glycine buffer, subsequently filtered with a 0.2 µm polytetrafluoroethylene (PTFE) syringe filter (VWR International S.r.l., Milano, Italy) to remove dye aggregates and further diluted in 1 × PBS to a concentration of 300 µM. The ThT dye stock was checked spectrophotometrically by measuring the absorbance at 412 nm (a molar extinction coefficient value of 36.000 M^−1^cm^−1^ was reported at this wavelength [32,48]). The mApoE-AuNP, bare AuNP, or free mApoE were added to 2.5 mL aliquots of the same Aβ + ThT solution to reach a final concentration of 1 µg/mL NP and 1.2 µg/mL mApoE, maintaining the same Aβ:NP and Aβ:mApoE stoichiometric ratios (1:3) as those used in AFM measurements. The disaggregation measurements were performed in parallel at 37 °C in fluorimetry quartz cuvettes, equipped with hermetic tip to avoid evaporation, employing the same Cary Ellipse fluorometer (Agilent, Santa Clara, CA, USA) used for the oligomerization studies. During the measurements, the solutions were gently stirred (200 rpm) using a magnetic stirrer (VEPL Scientifica S.r.l., Usmate Velate, Italy) embedded within the cell holder. Fluorescent excitation was at 450 nm and emission at 485 nm. The excitation and emission band-pass were set to 4 nm. All measurements were repeated on three different solutions.

## 5. Conclusions

Overall, these results show that mApoE can inhibit Aβ aggregation and promote its disaggregation in vitro. Interestingly, the effect of mApoE increases when concentrated on the AuNP surface, probably due to multivalency interactions between mApoE and Aβ. Therefore, the performance of anti-amyloidogenic molecules can be improved by modifying its clustering, and NP may be used to achieve this. 

## Abbreviations

Aβ	Amyloid-β
AD	Alzheimer’s disease
BBB	Blood brain barrier
AFM	Atomic force microscopy
TEM	Transmission Electron Microscopy
ThT	Thioflavin T
mApoE	Peptide CWG-LRKLRKRLLR
ApoE	Apolipoprotein E
AuNPDLS	Gold nanoparticlesDynamic Light Scattering
